# Cost-effectiveness of counseling and pedometer use to increase physical activity in the Netherlands: a modeling study

**DOI:** 10.1186/1478-7547-10-13

**Published:** 2012-09-24

**Authors:** Eelco AB Over, GC Wanda Wendel-Vos, Matthijs van den Berg, Heleen H Hamberg-van Reenen, Luqman Tariq, Rudolf T Hoogenveen, Pieter HM van Baal

**Affiliations:** 1 Centre for Prevention and Health Services Research, National Institute for Public Health and the Environment, P.O. Box 1, 3720, BA, Bilthoven, The Netherlands; 2 Centre for Public Health Forecasting, National Institute for Public Health and the Environment, P.O. Box 1, 3720, BA, Bilthoven, The Netherlands; 3 Expertise Centre for Methodology and Information Services, National Institute for Public Health and the Environment, P.O. Box 1, 3720, BA, Bilthoven, The Netherlands; 4 Institute of Health Policy and Management/Institute for Medical Technology Assessment, Erasmus University Rotterdam, Rotterdam, The Netherlands; 5 Present address: GlaxoSmithKline, P.O. Box 780, 3700, AT, Zeist, The Netherlands

**Keywords:** Economic evaluation, Prevention, Modeling, Counseling, Pedometer use, Physical activity, Primary care

## Abstract

**Background:**

Counseling in combination with pedometer use has proven to be effective in increasing physical activity and improving health outcomes. We investigated the cost-effectiveness of this intervention targeted at one million insufficiently active adults who visit their general practitioner in the Netherlands.

**Methods:**

We used the RIVM chronic disease model to estimate the long-term effects of increased physical activity on the future health care costs and quality adjusted life years (QALY) gained, from a health care perspective.

**Results:**

The intervention resulted in almost 6000 people shifting to more favorable physical-activity levels, and in 5100 life years and 6100 QALYs gained, at an additional total cost of EUR 67.6 million. The incremental cost-effectiveness ratio (ICER) was EUR 13,200 per life year gained and EUR 11,100 per QALY gained. The intervention has a probability of 0.66 to be cost-effective if a QALY gained is valued at the Dutch informal threshold for cost-effectiveness of preventive intervention of EUR 20,000. A sensitivity analysis showed substantial uncertainty of ICER values.

**Conclusion:**

Counseling in combination with pedometer use aiming to increase physical activity may be a cost-effective intervention. However, the intervention only yields relatively small health benefits in the Netherlands.

## Background

Lack of physical activity increases the risk of numerous adverse health conditions, including coronary artery disease, hypertension, stroke, insulin sensitivity, osteoporosis, and cancer [1]. In the Netherlands, it has been estimated that about 6% of total mortality can be attributed to physical inactivity [2]. In 2007, about 44% of the Dutch population aged 12 or above did not meet the recommendation to accumulate at least 30 minutes of moderate-intensity physical activity on at least five days of the week, and were classified as physically inactive (17%) or insufficiently active (27%) [3]. Therefore, increasing physical activity has the potential to reduce the burden of disease in the Netherlands considerably.

Recently, pedometers have become a popular tool for motivating people to engage in physical activity [4,5]. Pedometers are small, relatively inexpensive, devices worn at the hip to count the number of steps walked per day. Together with a calendar, diary, or daily log to keep record of and reflect on the amount of physical activity, the pedometer can be used to effectively increase daily physical activity [5]. A meta-analysis by Bravata et al. [4] of eight randomized controlled trials showed an average increase of 2491 (95% C.I. 1098 – 3885) steps per day among insufficiently-active outpatient adults and sedentary healthy adults randomly assigned to pedometer use for a mean period of 18 weeks (range: 3–104 weeks). These pedometer interventions, of which about two thirds included additional physical activity counseling, improved the patients’ risk factor profiles: on average, body mass index decreased by 0.38 kg/m^2^, systolic blood pressure by 3.8 mmHg and diastolic blood pressure by 0.3 mmHg.

Although pedometer use has been shown to be effective in increasing physical activity and improving health outcomes, little is known about its cost-effectiveness. To our knowledge, only two cost-effectiveness studies were published [6,7]. Using a simulation model, Cobiac and colleagues investigated the cost-effectiveness of pedometer use in the Australian population from a health care perspective. They reported that the use of pedometers in the Australian population would be dominant, i.e. it would be successful in averting disability adjusted life years and less costly compared to standard care. In this cost-effectiveness study, the pedometer intervention was applied through a community program, targeted at everyone aged 15 and over. Cobiac et al. assumed that 12.5% of the target population would start using a pedometer. They did not state how the pedometers would be distributed, and the direct intervention costs were calculated as a weighted average from each of the eight randomized controlled trials analyzed by Bravata et al. [4]. De Smedt and colleagues also reported that the Belgian community-based pedometer intervention “10,000 Steps Ghent” would be dominant. In their state-transition Markov model there was a direct link between increased walking and reduced risks of diabetes and cardiovascular and oncological life events. They modeled continuous implementation of the pedometer intervention.

In the present study, we used mathematical modeling to perform a cost-effectiveness analysis (CEA) of counseling in combination with pedometers in a primary care setting. The outcome of this CEA is expressed in the incremental cost-effectiveness ratio (ICER) - the ratio of incremental costs to incremental effects of the intervention - expressed in 2009 euros per quality adjusted life year (QALY) [8].

## Methods

### Two scenarios

We compared the scenario in which general practitioners (GPs) offer their patients that have been identified as being insufficiently active to use a pedometer (pedometer scenario) to the scenario of current practice. In the current practice scenario, no additional actions are taken with respect to the patients’ physical activity, assuming that their physical activity will only change because of aging. Table 1 shows the course of the pedometer scenario and the number of participants in each stage of the intervention. In 2008, the total Dutch population aged 20–65 years accounted for about 10 million people [9] and 7.27 million of them visited the GP at least once according to Statistics Netherlands [10]. We assumed that the percentage of GPs participating in the pedometer intervention would be equal to the percentage that opportunistically offers a minimal smoking-cessation intervention in the Netherlands, namely 35%-40% [11]. We assumed that this percentage would be equal to the percentage of the Dutch population having access to the pedometer intervention. Patients visiting a participating GP fill in a short physical activity questionnaire: the SQUASH, introduced by Wendel-Vos et al. [12]. Wendel-Vos et al. showed that 83% of the people that were offered a SQUASH, actually filled it in. The GP then scores the SQUASH, informs the patients about their physical-activity level, and offers a pedometer to those patients who are not sufficiently active, which is approximately 44% [3]. The patients further have to attend three follow-up sessions with the GP’s assistant to complete the pedometer intervention. No data is currently available as to how many patients would be willing to accept a pedometer and how many of those would participate in the follow-up sessions. Therefore, we assumed these last two parameters to be 50%, and applied large confidence intervals (CI) around them in the sensitivity analysis (see below). The pedometer scenario is assumed to increase the physical-activity levels of those patients completing the pedometer intervention by 2491 steps per day after one year, which is the average increase in steps found by Bravata et al. [4].

**Table 1:** Numbers of persons in successive stages of the pedometer scenario, and associated costs

	**Number of persons**	**Calculation**	**Type of cost**	**Cost per person**	**Total cost**
Dutch population aged 20–65 years visiting GP	7,270,000				
Visiting participating GP	2,726,000	7,270,000*0.375	Approaching patients, by GP assistant (1 min)	0.66	1,799,160
Screened with the SQUASH questionnaire	2,263,000	2,726,000*0.83	Checking physical-activity level, by GP (3 min)	6.66	15,071,580
Target population (those not norm active)	997,900	2,263,000*0.441	Counseling, by GP (10 min)	22.20	22,153,380
Receiving a pedometer	498,900	997,900*0.50	Pedometer with electronic diary	19.95	9,953,055
Participating in follow-up sessions	249,500	498,900*0.50	Three follow-up sessions, by GP assistant (10 min each)	19.80	4,940,100
Not changing physical-activity level:	243,600	Based on POLS data, and estimation of long-term effect (see Methods section)			
Inactive adults becoming semi-active:	1,300				
Inactive adults becoming norm active:	3,500				
Insufficiently active adults becoming norm active:	1,100				

### The RIVM chronic disease model

We used the RIVM Chronic Disease Model (CDM), a Markov-type state-transition model [13-15], to calculate the effects of the pedometer scenario compared to the current practice scenario, in terms of QALYs. The QALYs were calculated by attaching appropriate utility weights to each possible health state in the model. The CDM is a computer model that can simulate the incidence, prevalence, and mortality of diabetes, myocardial infarction, stroke, and colorectal and breast cancer because of physical inactivity among the Dutch population. The incidence and mortality of these diseases also depend on the presence of other diseases via relative risks. All data in the model are age and sex specific. For a fully detailed description of the RIVM CDM, see Hoogenveen et al. [14]. We discounted all future effects at 1.5%, as recommended in the Dutch guidelines for pharmacoeconomic research [16].

The scenario input of the CDM is a change in one or more risk factor distributions. The risk factor “physical activity” in the CDM consists of three discrete levels, analogous to the Dutch physical-activity guideline [17] (see below). We calculated the shift to more favorable levels of physical activity, due to additional steps in the pedometer scenario, on the basis of data from the annual General Public Health and Lifestyle Survey (Dutch acronym: POLS) from 2001 to 2007, conducted by Statistics Netherlands [10]. This shift was assumed to be the difference between two distributions over the physical-activity levels: 1) the distribution of the entire POLS data set, and 2) the distribution of a subgroup of people in the POLS data set as walking (in commuter traffic or at one’s leisure) an average 23 minutes more than the average of the entire POLS data set. With an average step pace of 106 steps per minute (see also [5,6]), 2491 correspond to 23 minutes of walking. The three levels of the Dutch physical activity guideline [17] are: 1) inactive (less than 30 minutes of at least moderately intense physical activity at all days of the week), 2) insufficiently active (30 minutes or more of at least moderately intense physical activity at one to four days of the week), and 3) norm active (30 minutes or more of at least moderately intense physical activity at five or more days of the week). We calculated the shift of the physical-activity levels separately for people younger and older than 55 years because “moderately intense” is defined differently for these two age groups [17].

Because the RIVM CDM input needs long-term effects as input, and because it is unknown how long physical-activity effects maintain, we used the estimation method of Jacobs-van der Bruggen et al. [18] to calculate the fraction of the effect that is maintained after 18 weeks. They estimated that long-term physical-activity effects for people with a life expectancy of almost 15 years would be 55% of the effect after one year. Taking into account that the life expectancy of our cohort of adults was almost 40 years and that the effect of 2491 steps occurred after 18 weeks, we estimated a long-term pedometer effect of approximately 25% of the additional 2491 steps, namely 623 steps, which corresponds to 6 minutes of walking. We performed a sensitivity analysis for the long-term effect with 12.5% and 50% of 2491 steps, corresponding to 3 and 12 minutes of additional walking per day, respectively.

The RIVM CDM also provided estimates for the costs of healthcare, including those in years lived longer [19]. As recommended in the Dutch guidelines for pharmacoeconomic research [16], we discounted all future costs at the annual percentage of 4.0%, and calculated the costs of the intervention using a so-called bottom-up method. Table 1 shows the unit costs and total intervention costs used. We calculated all costs in 2009 euros.

We used the probabilistic sensitivity analysis (PSA) to account for uncertainty in the input parameters of the model (see Table 2). We ran the model 100,000 times, varying all parameters simultaneously. For the incidence, prevalence, and mortality rates of each disease [20], we had coupled sets of random draws because they are dependent parameters.

**Table 2:** Uncertain model parameters and their properties

	**Distribution**	**Mean**	**95% CI**	**Reference**
Fraction of participating GPs	beta	0.375	0.056–0.789	[11]
Fraction filling in SQUASH	beta	0.83	0.54–0.99	[12]
Costs for scoring SQUASH	2.22 * (1+ 4*beta)	6.66	3.37–9.95	Expert opinion. Minimum one minute (digital scoring) to maximum five minutes (manual scoring, multiple sports)
Fraction accepting pedometer, and fraction completing follow-up sessions	beta	0.50	0.13–0.87	
Additional steps per day	normal	2491	1098–3885	[4]
Fraction of the effect sustained in the long term	beta	0.25	0.08–0.47	[18]
Incidence, prevalence, and mortality rates of each disease	Poisson and binomial			[20]

## Results

**Figure 1:**
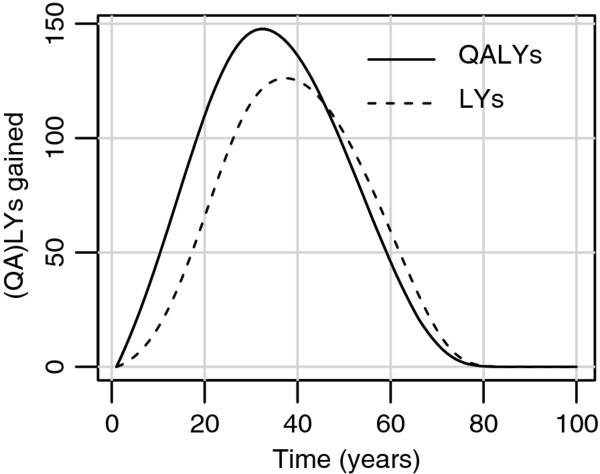
** Future effects of the pedometer scenario.** Life years (dashed line) and QALYs (solid line) gained in the pedometer scenario compared to the current practice scenario.

The pedometer scenario resulted in 4,600 life years or 5,500 QALYs gained compared to the current practice scenario. Figure 1 displays the amount of life years and QALYs gained as a function of future time. The intervention costs amounted to EUR 54.1 million, the healthcare costs (including costs in life years gained) amounted to EUR 7.1 million, making the total costs EUR 61.2 million. This resulted in an ICER of EUR 11,100 per QALY, and EUR 13,200 per life year.

**Figure 2:**
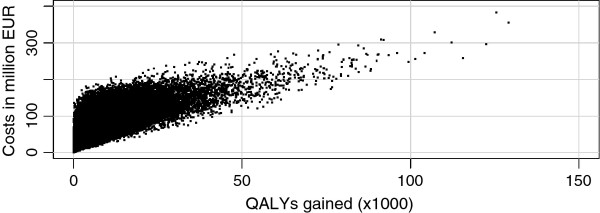
** Incremental costs and effects plane.** Costs versus effects for 100,000 simulations of the pedometer scenario compared to the current practice scenario (including health care costs in life years gained).

The sensitivity analysis for the long-term effect of additional walking per day showed an ICER of EUR 22,300 per QALY, and EUR 26,400 per life year for three extra minutes of walking. For 12 additional minutes of walking, the ICER was EUR 5,200 per QALY, and EUR 6,100 per life year. Figure 2 displays the total costs and total effects plane for the pedometer scenario compared to the current practice scenario, following from the PSA. Each dot represents a model run, with random values for the parameters mentioned in Table 2. The costs included those in life years gained, and the effects were expressed in QALYs. Table 3 shows the mean incremental life years and QALYs gained, health care costs incurred and the resulting ICER for the pedometer scenario compared to the current practice scenario.

**Table 3:** Estimates of total incremental costs and effects

	**Health care costs in life years gained**	
Life years gained		4,900 (200 – 21,700)
QALYs gained		5,800 (200 – 25,600)
Intervention costs (million EUR)		54.1 (7.3 – 124.8)
Future health care costs difference (million EUR)	Excluded	−16.5 (−72.5 – -0.7)
	Included	7.8 (0.3 – 34.7)
Total costs difference (million EUR)	Excluded	37.6 (0.2 – 96.6)
	Included	61.9 (8.1 – 149.4)
Euros per life year gained (EUR)	Excluded	7,600
	Included	12,500
Euros per QALY gained (EUR)	Excluded	6,500
	Included	10,600

Average costs and effects (95% CI) for the pedometer scenario compared to the current practice scenario. Costs discounted at 4.0% per year, effects (life years and QALYs gained) discounted at 1.5% per year.

**Figure 3:**
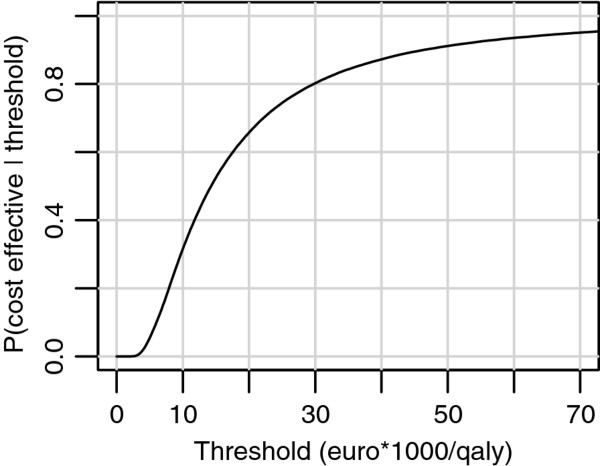
** Cost-effectiveness acceptability curve.** Probability of the pedometer scenario to be cost-effective compared to the current practice scenario as a function of cost-effectiveness threshold.

Figure 3 displays the cost-effectiveness acceptability curve for the pedometer scenario. Counseling in combination with pedometer use had a probability of 0.66 to be cost-effective when the threshold is EUR 20,000 per QALY gained. Since the Dutch informal threshold for cost-effectiveness of preventive interventions is EUR 20,000 per QALY, the pedometer scenario appeared to be cost-effective.

## Discussion

In this study, we have made some assumptions that need to be confirmed by further research. We used meta-analysis based results of the effect of pedometer use on the number of additional steps per day. However, the potential effect of pedometer use in the Dutch population is debatable. For the pedometer scenario, we used an additional 2491 steps per day after one year [4], but walking is already a substantial part of the daily physical activity pattern in the Netherlands [21]. It therefore remains to be seen if pedometer use in the Netherlands would indeed result in an additional 2491 steps per day. This may have led to an underestimation of the ICER. On top of that, the randomized controlled trials in Bravata et al. were mainly targeted at sedentary adults. The cohort of adults in our study is mostly not sedentary, and thus we may have overestimated the effects, also leading to an underestimation of the ICER. On the other hand, the additional 2491 steps found by Bravata et al. was a pooled effect of interventions with and without counseling, whereas our pedometer intervention always included counseling. This could mean that we underestimated the effects, and overestimated the ICER. We also had to make an assumption about the long-term effect of pedometers, which we estimated to be 25% of the effect after one year, namely 623 steps or 6 minutes per day. The accuracy of this estimation is unknown. If the long-term effect would amount to only three or less minutes of walking per day, the pedometer intervention would not be cost-effective.

A limitation of this study is that physical activity in the RIVM CDM is modeled via discrete classes: inactive, insufficiently active, and sufficiently active. Obviously, the real distribution of physical activity over the Dutch population is continuous, and even a single step per day more may have a (may it be minuscule) positive effect on health outcomes. Using discrete classes for physical activity implies a somewhat crude calculation of health outcomes.

In this study, we have chosen the health care perspective, focusing on health gains and health care costs but ignoring broader societal costs and consequences (higher labor productivity, less absenteeism, less environmental pollution, non-medical consumption in life years gained) of an increase in physical activity that fall outside the scope of the health care budget. This may have led to an overestimation of the ICER.

Comparing our results with the results of two previous economic evaluations, both Cobiac et al. [6] and De Smedt et al. [7] found the cost-effectiveness of their pedometer intervention to be dominant, i.e. health gain can be achieved while saving costs compared to current practice. A possible explanation for the more favorable result of Cobiac et al. [6] may be that the average proportion of inactive persons is much larger in the Australian than in the Dutch population (about 35% vs. 17%), which implies a higher possible health gain. De Smedt et al. [7] used much lower costs per person compared to our program costs: The total program costs per person accumulated to EUR 3.51 in the first year and EUR 0.23 in the second until fifth year. This is probably the main reason that their ICER was more favorable. Another difference between our and their study is that 65% of their target population group consisted of highly educated people. As prevention interventions are often more effective for high SES than low SES [22,23], the “10,000 Steps Ghent” program may have been more effective and more cost-effective than our pedometer intervention.

The cost-effectiveness of the pedometer intervention in the Netherlands in this study is less favorable than the cost-effectiveness of other types of physical activity interventions: an internet-based intervention program, a GP physical activity prescription program and a program to encourage more active transport all have a high probability of being cost-effective [6], and brief interventions in primary care [24], exercise referral [24], and mass media-based community campaign [6] have found to be even dominant. This difference is probably not due to the nature of the intervention, but rather because 56% of the Dutch population aged 20–65 already satisfies the Dutch guidelines for the healthiest level of physical activity.

## Conclusions

In summary, counseling in combination with pedometer use in order to increase physical activity appeared to be cost-effective in the Netherlands From a health care perspective. The resulting ICER was EUR 11,100 per QALY gained, which is below the Dutch informal threshold of EUR 20,000 per QALY gained. The PSA showed that the pedometer scenario had a probability of 0.66 to be cost-effective at this threshold.

However, since the intervention only yields relatively small health benefits in the Netherlands, it may be more worthwhile to allocate scarce resources to more cost-effective physical-activity interventions. Furthermore, since there is a lack of evidence regarding the extent to which pedometer users adhere to their increased activity levels, the uncertainty of the ICER is substantial: The sensitivity analysis showed a highly skewed distribution of ICERs, some of them even larger than a million Euros per QALY. Therefore, some caution is recommended with respect to the cost-effective nature of the pedometer intervention.

## Abbreviations

CEA: Cost-effectiveness analysis; CI: Confidence interval; GP: General practitioner; ICER: Incremental cost-effectiveness ratio; PSA: Probabilistic sensitivity analysis; QALY: Quality adjusted life year; RIVM CDM: Chronic Disease Model of the National Institute of Public Health and the Environment (Dutch acronym: RIVM).

## Competing interests

The authors declare that they have no competing interests.

## Authors’ contributions

EO helped design the study, performed the analyses and the modelling, and finished the manuscript. LT designed the study and drafted the manuscript. RH participated in the modelling. WW, MB, HH and PB participated in the design of the study and helped to draft the manuscript. All authors revised, read and approved the final manuscript.
